# The clinicopathological features and prognosis of signet ring cell carcinoma of the esophagus: A 10-year retrospective study in China

**DOI:** 10.1371/journal.pone.0176637

**Published:** 2017-05-09

**Authors:** Lei Chen, Xi Liu, Linggen Gao, Rong Wang, Dewei Gao, Dongyu Bai

**Affiliations:** 1 Department of Thoracic Surgery, General Hospital of Chinese People's Liberation Army, Beijing, China; 2 Department of Comprehensive Surgery, General Hospital of Chinese People's Liberation Army, Beijing, China; 3 Department of Pathology, the First Affiliated Hospital of Xiamen University, Province Fujian, Xiamen, China; Chinese Academy of Medical Sciences Institute of Basic Medical Sciences, CHINA

## Abstract

**Objectives:**

The aim of this study was to analyze the clinicopathological features and prognosis of esophageal signet ring cell (SRC) carcinoma in China.

**Methods:**

Patients with poorly differentiated adenocarcinoma were identified in two hospitals from January 2006 to June 2016. The patients were divided into three groups according to component of SRCs: SRC≥50% group, SRC < 50% group and non-SRC poorly differentiated adenocarcinoma group.

**Results:**

Fifty-seven patients had carcinoma (SRC≥50%), and 79 patients had tumors containing <50% SRCs, and 535 patients was in non-SRC poorly differentiated adenocarcinoma group. There were no significant differences among the three groups in clinicopathological characteristics. Patients in SRC≥50% group had a lower overall survival rate (at 3-year 37.6%versus71.1%; at 5-year 0% versus 43.3%; p<0.001) compared with the control group. Even survival outcome of patients in SRC < 50%was inferior to that of in control group (at 3-year 53.0%versus71.1%; at 5-year 25.9% versus 43.3%; p<0.001). Female sex, large tumor size and increasing TNM stage were independent prognostic factors for SRC ≥50% esophageal carcinoma patients.

**Conclusions:**

The incidence of esophageal SRC carcinoma is relatively rare and the worst outcome is observed in the SRC≥ 50% group. It is necessary to explore new therapeutic modalities to achieve further improvements in the clinical outcome of these patients.

## Introduction

Esophageal carcinomas have usually been found to be squamous-cell carcinomas [1, 2]. During the past 20 years, the incidence of squamous-cell carcinomas has decreased in the United States, while the rate of adenocarcinoma has increased [3]. Smoking and obesity may account for some of these changes[1]. Adenocarcinoma of the esophagus has a poor prognosis. Signet ring cell (SRC) carcinoma is a form of adenocarcinoma which was defined by the World Health Organization classification as those where more than 50% of the tumor consisted of isolated or small groups of malignant cells containing intracytoplasmic mucins[4]. The incidence of esophageal signet ring cell carcinomas is estimated to range from 3.5% to 5% for all esophageal malignancies in western studies [5–7]. Although these studies have reported on the incidence, clinicopathological features and prognosis of signet ring cell carcinoma of the esophagus and the gastroesophageal junction, it has not been well studied in the Asian population. Most SRC carcinoma in the gastroesophageal junction is originated from stomach, the clinicopathological features and prognosis of SRC carcinoma originated from the esophagus (excluding gastroesophageal junction) is not clear.

In this study, we sought to examine the constituent ratio of adenocarcinoma in all esophageal malignancies, review the clinical characteristics, treatment, and prognosis of cases with primary SRC carcinoma of the esophagus (not including carcinoma in the gastroesophageal junction) and compare them with those of adenocarcinomas without documented signet ring cell features.

In general, the prognosis of patients with SRC carcinoma of any organ site is poor. However, a large-volume study from the United States demonstrated that after adjusting for age, SRC does not necessarily portend a worse prognosis [8]. The results of these studies were not consistent. These discrepancies can be partly explained by the methodology and design variations of each study, the heterogeneity of non-SRC groups according to tumor differentiation. SRC carcinoma is poorly differentiated adenocarcinoma and confers worse prognosis than well-to-moderately differentiated adenocarcinoma[9]. In this aspect, in order to analyze the clinicopathologic characteristics and prognosis of esophageal SRC carcinoma, poorly differentiated non-SRC adenocarcinoma was used as control group.

## Patients and methods

A total of 10,461 cases with esophageal carcinomas underwent elective surgical resection in the Department of thoracic surgery Chinese PLA General Hospital and Department of thoracic surgery the First Affiliated Hospital of Xiamen University from January 2006 to June 2016. Patients who had histologically confirmed adenocarcinoma of the esophagus and histologically proved containing SRCs were identified by examination of pretreatment biopsy and final pathology reports. Adenocarcinoma was also classified as well (well-formed glands), moderately, or poorly differentiated (highly irregular glands that are recognized with difficulty) according to the WHO classification. Data were retrieved from operative and pathological reports, and follow-up data were obtained by phone, outpatient and clinical databases.

In order to evaluate the impact of the presence of SRCs, the cases were divided into 3 groups on the basis of the definition of the WHO: SRC ≥50% group, SRC < 50% group and non-SRC poorly differentiated adenocarcinoma. Non-SRC poorly differentiated adenocarcinoma group served as control group.

Diagnostic investigations routinely included a history taking, physical examination, routine laboratory tests, a barium study and an esophagi-gastro-duodenoscopy with biopsies, a neck and thoracoabdominal CT scan, selective endoscopic ultrasound evaluation, and external ultrasonography of the neck.

### Treatment

All of the included patients underwent elective esophagectomy. The type of resection was dependent on location of tumor and surgeon preference. The location of the upper, the middle and the lower esophageal carcinoma is at 20–25 centimeter (cm), 25–30 cm and 30–40 cm from the incisor teeth respectively. Open Ivor-Lewis included a two-field lymph node dissection through a laparotomy and muscle-sparing right thoracotomy. None of the McKeown procedures included a cervical field node dissection. Minimally invasive procedures were a combination of complete minimal access cases in abdomen and chest plus the hybrid cases for which either the abdominal or thoracic component of the operation was performed in the standard, open fashion. None of the included patients underwent radiotherapy or chemotherapy before surgery, and none had prior malignant disease or metastatic spread on routine examination before surgery. Patients with positive lymph node metastasis or positive margin in a resected specimen, postoperative radiotherapy or adjuvant chemotherapy were administered.

### Histopathological analysis

**Fig 1:**
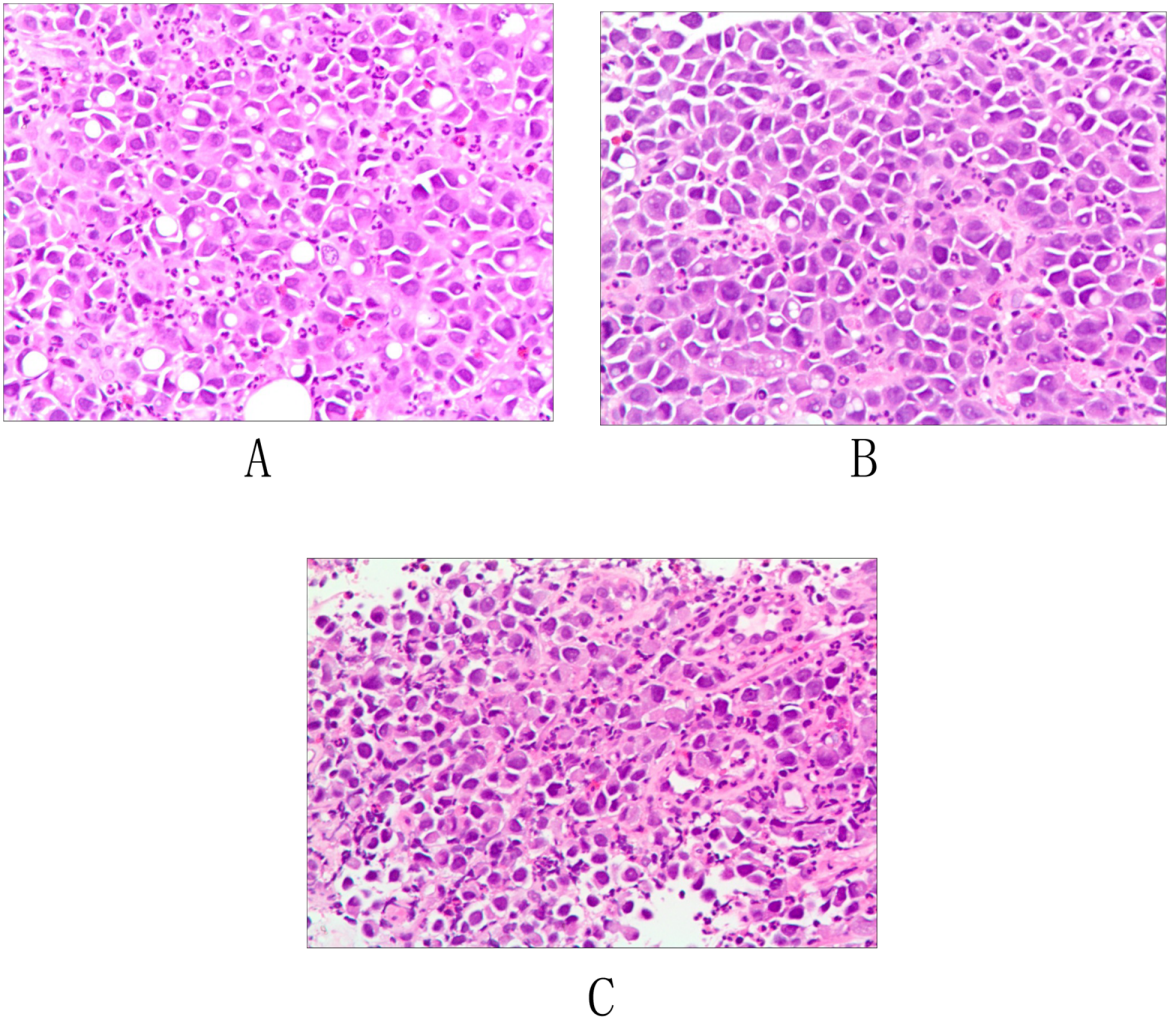
Hematoxylin and eosin—Stained section (200 magnification) of a biopsy specimen showing (A) SRC≥50% and (B) SRC<50%, and (C) poorly differentiated adenocarcinoma (reference group).

All specimens were blindly reevaluated by the same experienced pathologist (Bai DY) specifically for the purpose of this study looking for presence of SRC, percentage of SRC volume as compared with the total volume of tumor, location of the primary lesion, depth of invasion, margin status, and lymph node metastases. Staging was determined according to the seventh edition of the American Joint Committee on Cancer system for esophageal carcinoma[10]. The WHO pathology classification was then applied to form the 2 study groups: carcinomas containing more than 50% SRCs (SRC ≥50%) (Fig 1A) and carcinomas containing less than 50% SRCs (SRC < 50%)(Fig 1B). Poorly differentiated adenocarcinoma without SRCs group served as control group (Fig 1C).

Microscopic radicality with a tumor-free resection margin is defined as R0. With R1 there is microscopic, and with R2 macroscopic residual tumor. Examples of R2 are tumor perforation, residual non-resectable tumor and/or metastasis-infiltrated lymph nodes.

### Ethical considerations

This was a retrospective study undertaken in the Department of thoracic surgery Chinese PLA General Hospital and Department of thoracic surgery the First Affiliated Hospital of Xiamen University from January 2006 to June 2016. Data were retrieved from operative and pathological reports, and follow-up data were obtained by phone, outpatient and clinical databases. Written informed consent was obtained from the patients or the patients’ close relatives. The study was approved by the Chinese PLA General Hospital / First Affiliated Hospital of Xiamen University Research Ethics Committee. All study methods were performed in accordance with the relevant guidelines and regulations.

## Statistical analysis

Data are reported as the mean±SD for continuous variables and as frequencies and percentages for categorical variables. Continuous variables were compared by Student t test for normally distributed values. Differences in percentages were evaluated using the Chi-square test. The survival time was calculated from the start of treatment to the point of death or last follow-up. We analyzed prognostic factors by Cox’s hazard regression model, with the entry factors of age (60 years versus 60 years), gender, location of the primary lesion, length of the primary lesion (5 cm versus 5 cm), pTNM stage. Survival curves during follow-up were plotted by the Kaplane Meier method. Significance was considered to be present for values of p<0.05. A commercial statistical software package (SPSS for Windows, version 17.0; SPSS, Chicago, IL) was used for data analysis.

## Results

### Patient demographics

**Fig 2:**
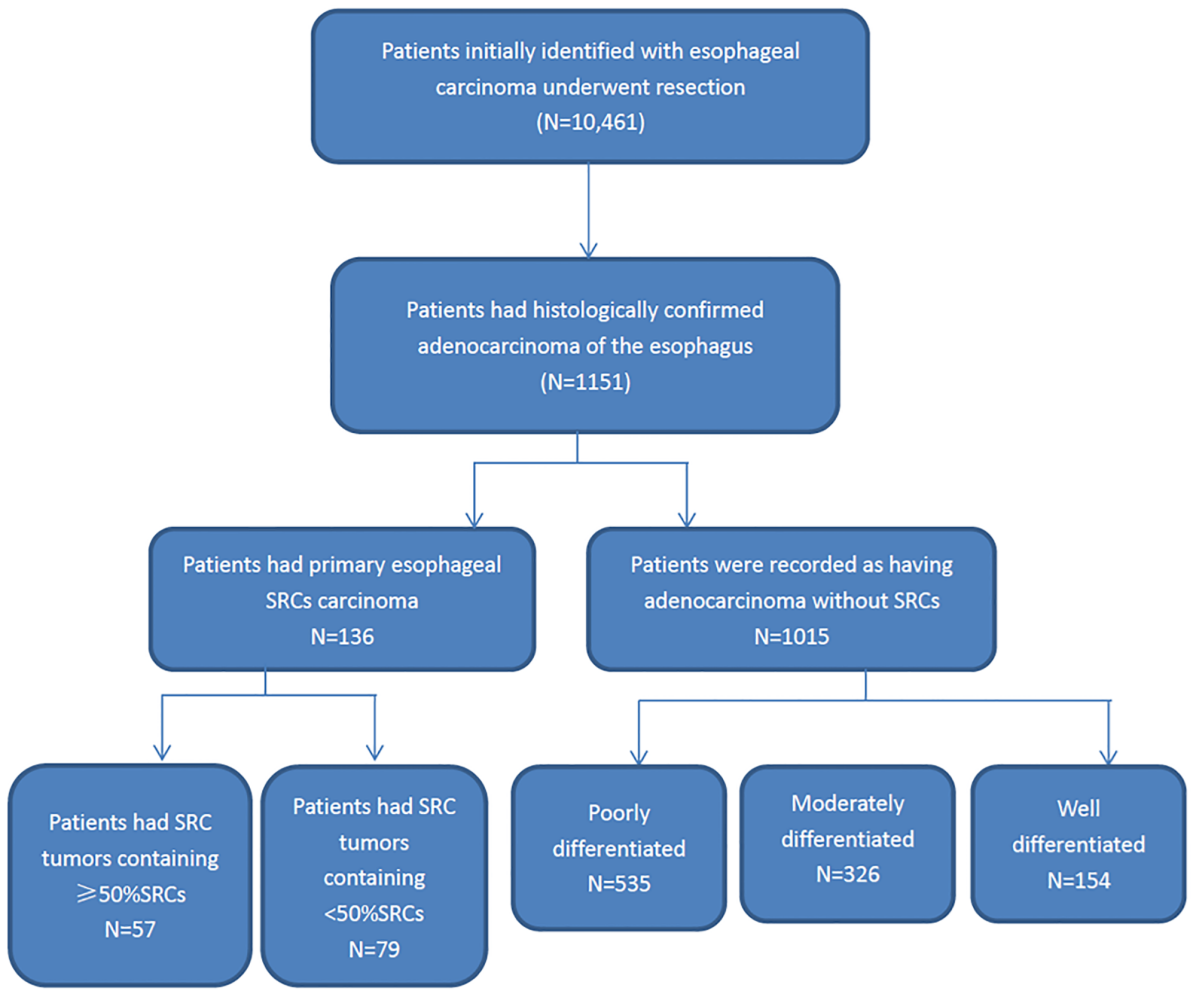
Consort diagram.

Of the 10,461 patients initially identified, 1151(11.01%)patients had histologically confirmed adenocarcinoma of the esophagus. Fifty-seven(0.55%)patients had primary esophageal SRCs carcinoma (SRC≥50%), and 79(0.76%)patients had tumors containing <50%SRCs, and 1015 (9.71%)patients were recorded as having adenocarcinoma without SRCs. Of these patients having adenocarcinoma without SRCs, 154 (1.48%) were well differentiated, 326 (3.12%) were moderately differentiated, and the remaining 535 (5.12%) were poorly differentiated (Fig 2). The clinical features of patients in the signet ring cell adenocarcinoma group and the reference group (poorly differentiated adenocarcinoma) are shown in Table 1. The esophageal SRCs carcinoma (SRC≥50%) group consisted of 49 men and 8 women with an average age of 58.1 years. Patients’ ASA grade was I or II in 78.9% of the cases. Pretreatment weight loss affected 22.2% of the patients. There were no statistically significant differences among the three comparison groups in demographic characteristics such as age, sex, BMI, family history of esophageal carcinoma and comorbid conditions.

**Table 1:** Characteristics of the patients according to the therapy group.

Variables	SRC≥50%,(n = 57)	SRC<50%,(n = 79)	SRC (n = 136)	PDA(n = 535)	P value (PDA vs SRC)	P value (SRC≥50% vs SRC<50%)
Age (years)	58.1±7.7	59.5±8.4	58.9±8.0	60.6±8.7	0.364	0.395
Sex (Male),n (%)	49(85.9)	67(84.8)	116(85.3)	420(78.5)	0.093	1.000
BMI, kg/m2, median (IQR)	22.5(19.5–25.7)	21.9(19.0–26.5)	22.1(19.1–26.5)	23.1(21.0–26.8)	0.295	0.368
Pretreatment weight loss, n (%)	18(31.6)	28(35.4)	46(33.8)	170(31.8)	0.681	0.715
Family history	9(15.8)	11(13.9)	20(14.7)	67(12.5)	0.478	0.809
Comorbid conditions						
History of gastroesophageal reflux disease	38(66.6)	59(74.7)	97(71.3)	363(67.9)	0.470	0.340
ASA grade						
I	15(26.3)	21(26.6)	36(26.5)	137(25.6)	0.827	1.000
II	30(52.6)	39(49.4)	69(50.7)	258(48.2)	0.632	0.731
III	12(21.1)	19(24.1)	31(22.8)	140(26.2)	0.443	0.836
Diabetes mellitus, n (%)	10(17.5)	16(20.2)	26(19.1)	105(19.6)	1.000	0.826
Coronary artery disease, n (%)	8(14.0)	9(8.9)	17(12.5)	66(12.3)	1.000	0.794
COPD/emphysema, n (%)	12(21.0)	11(13.9)	23(16.9)	82(15.3)	0.692	0.354
Chronic renal insufficiency, baseline Cr > 2 mg/dL or HD, n (%)	3(5.2)	2(2.5)	5(3.7)	9(1.7)	0.174	0.649
Smoking history						
Current	9(15.8)	10(12.7)	19(14.0)	72(13.5)	0.889	0.624
Former	25(43.9)	31(39.2)	56(41.2)	288(53.8)	0.009	0.601
Never	23(40.3)	38(48.1)	61(44.9)	175(32.7)	0.009	0.388

BMI, indicates body mass index; HD, hemodialysis; PDA, poorly differentiated adenocarcinoma; p*,

### Clinical pathologic characteristics

The clinical pathologic characteristics of patients in the SRC adenocarcinoma groups(SRC≥50% and SRC<50%) and the reference group (poorly differentiated adenocarcinoma) are shown in Table 2. Most tumors (75.4%) were in the middle and lower third of the esophageal and the clinical stage II-III was observed in 87.7% of the SRC≥50% group. R0 resection rates was 86.0%, and positive margin was observed in 14% in the SRC≥50% group with positive proximal margin in 6 (11.5%), distal margin in 5 (8.8%), lateral margin in 5(8.8%) and longitudinal margin in 4 (7%). There were no statistically significant differences between groups (SRC vs. non-SRC group; SRC≥50% vs SRC<50% group) in clinical pathologic characteristics such as clinical stage, length, positive margin and lymph nodes harvested.

**Table 2:** Perioperative and histopathological variables of the resected specimen.

Variables	SRC≥50%(n = 57),	SRC<50%(n = 79)	SRC,n = (136)	PDA(n = 535)	P value (PDA vs SRC)	P value (SRC≥50% vs SRC<50%)
Resection						
R0	40(70.2)	61(77.2)	101(74.3)	440(82.4)	0.039	0.428
R1	12(21.1)	12(15.2)	24(17.6)	53(9.9)	0.016	0.495
R2	5(8.8)	6(7.6)	11(8.1)	42(7.8)	0.861	1.000
Location						
Upper	8(14.0)	13(16.4)	21(15.4)	86(16.1)	1.000	0.812
Middle	15(26.3)	18(22.8)	33(24.3)	107(20.0)	0.288	0.688
Lower	28(49.1)	36(45.6)	64(47.1)	303(56.6)	0.054	0.729
Two-third and more	6(10.5)	12(15.2)	18(13.2)	39(7.2)	0.037	0.609
Length						
≤5 cm	37(64.9)	55(69.6)	92(67.6)	316(59.1)	0.077	0.582
>5 cm	20(35.1)	24(30.4)	44(32.3)	219(40.9)	0.077	0.582
Adjuvant treatment	17(29.8)	18(22.8)	35(25.7)	95(17.8)	0.039	0.428
Resection						
Proximal margin(-)	51(89.5)	70(88.6)	121(89.0)	477(89.2)	1.000	1.000
Distal margin(-)	52(91.2)	73(92.4)	125(91.9)	485(90.7)	0.740	1.000
Longitudinal margin	53(93.0)	72(91.1)	125(91.9)	488(91.2)	0.866	0.761
Lateral margin	52(91.2)	74(93.7)	126(92.6)	497(92.9)	0.854	0.742
Lymph nodes harvested(n)	28.5±12.1	25.8±10.5	26.9±11.4	26.3±13.6	0.673	0.792
TNM stage						
I	7(12.3)	9(11.4)	16(11.8)	53(9.9)	0.528	1.000
II	18(31.6)	30(38.0)	48(35.3)	214(40.0)	0.327	0.472
III	32(56.1)	40(50.6)	72(52.9)	268(50.1)	0.566	0.602

### Survival

**Fig 3:**
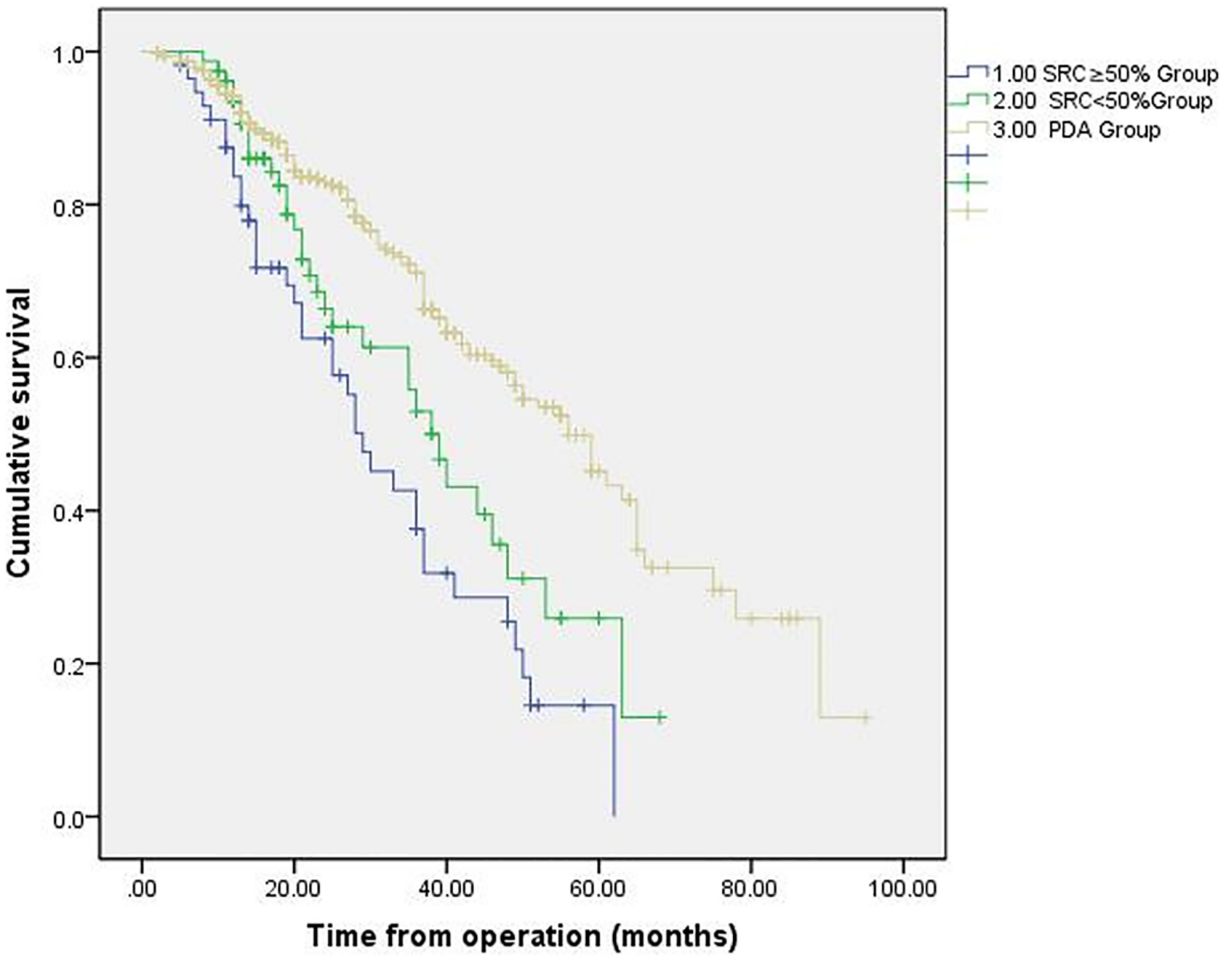
Kaplan-Meier survival curves by the presence of SRCs.

The overall survival curves for all patients from the SRC groups (SRC≥50% and SRC<50%) and patients from the reference group were shown in Fig 3. The median overall survival duration for patients in the SRC≥50% group was 29 months (95% confidence interval [CI]: 21 to 36), which was significantly shorter than that observed in the SRC<50% group (39 months, 95% CI: 33 to 44) and the reference group (56 months, 95% CI: 48 to 63). Patients in the SRC≥50% group had a lower overall survival rate (at 1-year 83.7%versus 94.1%, at 3-year 37.6%versus71.1%; at 5-year 0% versus 43.3%; p<0.001) compared with the reference group. Even survival outcome of patients in the SRC<50% group was inferior to that of the reference population (at 1-year 93.5%versus 94.1%, at 3-year 53.0%versus71.1%; at 5-year 25.9% versus 43.3%; p<0.001). Examination of the association of individual variables with overall survival showed that female sex, large tumor size and increasing TNM stage were independent prognostic factors for patients with SRC ≥50% esophageal carcinoma (Table 3).

**Table 3:** Univariate analysis of the 57 patients with primary esophageal carcinoma containing ≥50%SRCs.

Variables	N	Survival Rate(%) 1 year	Survival Rate(%) 2 years	Survival Rate(%)3 years	Survival Rate(%)5 years	Median survival time, months (95%CI)	P	Univariate Analysis Variables on late death HR(95%CI)	P
Gender									
Male	4	91.6	73.7	49.3	19.3	36.0(27.2–44.7)	<0.0001	1	
Female	8	62.5	20.8	0	0	13.0(4.65–21.3)		2.71 (1.85–3.04)	<0.001
Age(years)									
<60	2	88.0	72.6	42.4	14.0	30.0(16.0–44.0)	0.404	1	
≥60	2	89.4	64.0	44.3	11.1	29.0(16.3–41.7)		1.01 (0.90–1.37)	0.17
Location									
Upper	8	85.7	68.6	45.7	0.0	36.0(5.88–66.1)	0.271	1	
Middle	1	86.2	71.1	55.3	27.6	37.0(24.9–49.1)		0.97(0.89–1.52)	0.70
Lower	2	89.0	62.6	29.0	0.0	27(21.6–32.5)		1.25(1.05–2.15)	0.02
Two-third and more	6	66.7	44.4	22.2	0.0	29(22.0–36.0)		1.20(1.03–2.19)	0.03
Length									
<5 cm	3	93.8	83.4	51.7	21.7	37.0(25.1–48.9)	0.000	1	
≥5 cm	2	67.9	28.2	14.1	0.0	29.0(22.0–36.0)		1.26(1.13–1.90)	0.03
TNM stage									
I	7	85.7	71.4	71.4	57.1	49.9(36.3–63.5)	0.000	1	
II	1	94.4	87.2	44.0	22.0	36.0(24.7–47.2)		1.53(1.15–2.16)	0.006
III	3	73.6	41.6	23.1	0.0	19(12.6–25.4)		4.05(3.35–9.91)	<0.001

CI, confidence interval; HR, hazard ratio.

## Discussion

In the present study, the frequency of SRC carcinoma of the esophagus was 0.55% (57/10,461). We have shown that primary esophageal SRCs carcinoma (SRC≥50%) have a much worse cancer-specific prognosis than poorly differentiated adenocarcinoma without SRCs (overall 5-year survival of 0% vs 57%, respectively; P<0.001). And survival outcome of patients in the SRC<50% group was inferior to that of the reference population. Our findings also indicate that female, tumor length≥5 cm and increased TNM stage were independent risk factors for the poor prognosis in esophageal SRCs carcinoma.

SRC carcinoma is a rare histologic variant of esophageal adenocarcinoma that has been recently increasing reported in the western countries[7]. Most of the published data reported the SRC carcinoma of the esophagus and esophagogastric junction [11–15]. Literature on the clinical characteristics and long term outcome of the esophagus SRC carcinoma focused on Asian population and the SRC carcinoma of the esophagus (excluding esophagogastric junction) is limited. In the present study, SRC histology was identified in 136 of 1015 patients with esophageal adenocarcinoma (13.4%). This was in line with recent report by Nafteux PR[6], which was focused on western population.

SRC carcinoma may be occurred in a variety of organs, including urinary bladder[16], prostate[17], the stomach[18], colon[19, 20], lung[21], and breast[22]. In general, the prognosis of patients with SRC carcinoma of any organ is poor[16, 19–24]. Our findings revealed that the median overall survival time of patients with esophageal SRCs carcinoma in our study was 29 months, which was much shorter than the 56 months in regular adenocarcinomas with the same stage. Based on our results, we confirmed that the existence of signet-ring cells may be a negatively prognostic maker regardless the percentage of SRC component.

Surgical resection is the primary therapy. The incidence of positive margins in SRC group was higher than that in the reference group, and this was consistent with the previous study[15]. The proportion of patients with R1 resection (17.6%vs9.9%, respectively; P = 0.016) and with tumor located more than two thirds of the esophageal (13.2%vs7.2%, respectively; P = 0.037) was higher in the SRC group as compared with the PDA group. This could possibly explain the lower survival in the SRC group than in the PDA group. A higher proportion of positive margins in the SRC group indicate that these patients may need to be resected a wider margin. And patients with positive margin in a resected specimen, postoperative radiotherapy or adjuvant chemotherapy were administered.

There was no significant difference among the three groups in the TNM stage at the time of diagnosis which was not consistent with previous investigation [15]. Early-stage esophageal SRC carcinomas are found to have better outcomes.

To our knowledge, this study was the first to analyze clinical parameters and long term outcome of patients with SRC carcinoma of the esophagus in Asia. However, our study had some limitations. Firstly, this study is limited by its retrospective nature, which may lead to missing data and may introduce bias. Secondly, the main problem with studying signet ring cell esophageal adenocarcinoma is its relative rarity, thus limiting the statistical power of such studies. Thirdly, the present study includes only surgically treated patients, excluding patients with metastasis at presentation or patients not sent for surgical resection due to comorbidities or other factors.

In summary, the incidence of SRC carcinoma of the esophagus is relatively rare and our study confirmed that patients with SRC carcinoma of the esophagus have a worse prognosis than reference group when treated by primary surgery. Surgical resection is the primary treatment, but the prognosis is poor. Female sex, large tumor size and increasing TNM stage portended worse prognosis are independent prognostic factors. It is necessary to achieve further improvements in the clinical outcome of patients with such tumors by developing new therapeutic modalities.
